# Phase-contingent resilience effects in multilingual medical students: a cross-sectional examination of student demands-resources theory

**DOI:** 10.3389/fmed.2026.1775910

**Published:** 2026-03-16

**Authors:** Sadia Qazi, Siyaan Yasser Qureshi, Eshal Atif, Muhammad Atif Mazhar, Ismail Mohammed, Mahdi Ameer, Akef Obeidat

**Affiliations:** 1 Department of Anatomy, College of Medicine, Alfaisal University, Riyadh, Saudi Arabia; 2 College of Medicine, Alfaisal University, Riyadh, Saudi Arabia

**Keywords:** clinical training, cross-sectional study, language proficiency, multilingual medical education, psychological resilience, student engagement

## Abstract

**Introduction:**

The Student Demands–Resources (SDR) theory proposes that psychological and environmental resources may become more relevant as situational demands increase. This mechanism remains underexamined in medical students. We assessed whether resilience and language proficiency were associated with academic engagement across training phases in a multilingual medical cohort.

**Methods:**

We surveyed 422 medical students at an international medical school (May–September 2024). Hierarchical multiple regression modeled academic engagement (UWES-9S) as a function of psychological resilience (BRS), Arabic language proficiency, clinical training phase, prior residence, and social support (DSSI). The Resilience × Clinical Phase interaction was specified *a priori*; other interactions were exploratory.

**Results:**

The main-effects model explained 52.8% of variance in engagement (*R*^2^ = 0.528). Resilience (*β* = 0.418, *f*^2^ = 0.370), sex (*β* = −0.410, *f*^2^ = 0.356), and Arabic proficiency (*β* = 0.370, *f*^2^ = 0.267) showed the largest effects. The resilience–engagement association was stronger in clinical than preclinical students (standardized *β*: 0.630 vs. 0.291; interaction *β* = 0.339, *p* < 0.001, *f*^2^ = 0.129). Female students showed lower overall engagement, with differences concentrated in specific dimensions. Social support showed small positive associations across dimensions. Sensitivity analysis excluding prior residence yielded near-identical estimates and unchanged inference (reduced-model *R*^2^ = 0.525; Δ*R*^2^ = −0.003).

**Conclusion:**

These hypothesis-generating findings are consistent with phase-contingent resource–engagement associations in SDR theory within medical education. The cross-sectional design and indirect demand proxy do not support causal inference.

## 1 Introduction

Medical student engagement, comprising vigor (emotional energy), dedication (sense of meaning), and absorption (focused immersion), is a robust predictor of academic performance, professional development, and long-term physician well-being (1, 2). Conversely, disengagement is associated with burnout, compassion fatigue, and reduced clinical satisfaction, with declines most consistently observed during transitions into clinical training (3, 4). These transitions represent critical vulnerability points in medical education, where increasing demands coincide with declining student engagement.

### 1.1 The incomplete picture: why individual resources are not enough

Psychological resilience is frequently promoted as a protective factor in high-stress professions and has been shown to correlate positively with engagement among medical students (5, 6). However, resilience alone explains only a modest proportion of the variance in engagement outcomes (7), suggesting that individual psychological capacity is insufficient as a standalone explanation for engagement.

A key limitation of much existing work is the implicit assumption that individual resources operate independently of the context. In practice, resources do not function in a vacuum; their effectiveness depends on their alignment with situational demands and available contextual support. When this alignment is ignored, estimates of resource effects may be diluted or misleading. This limitation is particularly salient in medical education, where demand intensity varies sharply across training phases; however, engagement models rarely explicitly account for this variation.

This context dependency of resources becomes even more complex in educational settings, where students face compounded demands, such as multilingual medical training environments, where linguistic, clinical, and cultural demands converge.

### 1.2 The context: multilingual medical education as a high-demand environment

An increasingly common educational model combines English-medium instruction with non-English clinical practice, which has been implemented in more than 40 countries and affects over 25,000 international medical students annually (8, 9). Students in these programs encounter structural and linguistic demands that are absent from monolingual curricula. They must master biomedical knowledge in English while communicating with patients in local languages and navigating culturally embedded clinical interactions (10, 11).

Despite the centrality of language to clinical participation, language proficiency is often treated as a fixed demographic attribute rather than a functional learning resource. Emerging work on translanguaging conceptualizes flexible language use as a teachable professional competency that supports meaning-making and communication in complex clinical settings (12, 13). Nevertheless, language remains largely peripheral in engagement research and is typically included, if at all, as a control variable rather than a theoretically relevant predictor.

Understanding how linguistic resources interact with psychological resources under varying demand conditions requires a theoretical framework that explicitly models the demand-resource dynamics.

### 1.3 Theoretical gap: why student demands–resources theory matters

To address these limitations, this study applies the Student Demand Resources (SDR) theory, adapted from the Job Demands Resources (JDR) model (14, 15). SDR theory conceptualizes engagement as a dynamic balance between situational demands and the resources available. Demands require sustained effort and deplete psychological capacity (e.g., clinical workload and performance pressure), whereas resources facilitate goal attainment, reduce perceived demands, and promote growth (e.g., resilience, social support, language proficiency, and environmental familiarity).

Crucially, SDR theory makes a specific prediction that remains largely untested in medical education: resource-engagement associations are phase-contingent, with resource effects strengthening as demand intensity increases. Under this framework, resilience may exert modest effects during lower-demand preclinical training but become substantially more consequential during clinical phase training, when responsibility, uncertainty, emotional exposure, and linguistic demands converge.

This mechanism has several important implications. It reframes resilience and language proficiency as context-sensitive resources rather than universally effective traits, clarifies why intervention timing matters, and explains why prior studies may have underestimated resource effects by averaging across training phases in which demands differ fundamentally. Multilingual medical education provides an ideal context for testing this mechanism because students experience both a well-defined transition in demand intensity (from preclinical to clinical) and compounded resource requirements (psychological resilience plus language proficiency).

### 1.4 Knowledge gap and research questions

Despite extensive research on resilience and increasing attention to multilingual competence in medical education (16–18), no prior study has directly tested whether resource-engagement associations vary according to demand intensity across the medical training phases. This omission is striking, given that clinical transition is consistently identified as a high-demand period (3), and theoretical models predict that resource effectiveness should depend on the severity of stressors.

Accordingly, this study addresses the following primary research question:

Do the associations between resilience, language proficiency, and academic engagement differ between the preclinical and clinical training phases, as predicted by SDR theory?

Secondary questions examined the relative contributions of resilience, language proficiency, contextual familiarity, and social support to engagement, as well as whether engagement differed by gender and through which dimensions these differences manifested.

Based on SDR theory, we hypothesized that: (H1) resilience and language proficiency independently predict engagement, with resilience showing the largest effect; (H2) the resilience–engagement association is stronger during the clinical phase than during the preclinical phase; (H3) language proficiency and prior environmental familiarity contribute to engagement beyond resilience; and (H4) social support enhances engagement, potentially in dimension-specific ways.

We operationalized demand intensity through training phase (preclinical Years 1–2 vs. clinical Years 3–4), based on evidence that clinical training involves higher workload, patient responsibility, emotional exposure, and performance pressure (3). However, training phase is an indirect proxy: it does not measure experienced demands directly, and students within the same phase may experience heterogeneous demand levels. Therefore, this study tests whether resource-engagement associations differ by training phase, a pattern we term “phase-contingent associations” which would be consistent with SDR’s demand-moderation mechanism but cannot definitively establish that demand intensity itself moderates resource effects. Alternative explanations (e.g., selection effects, developmental maturation, or unmeasured confounders) cannot be ruled out with this cross-sectional design. Findings should be interpreted as hypothesis-generating evidence warranting replication with direct demand measurement (e.g., validated workload scales, clinical hours, perceived stress indices) and longitudinal designs.

This study tests these predictions in a multilingual medical student population in a high-income Gulf context where English-medium instruction is paired with Arabic-dominant clinical practice. This setting offers a naturalistic context for examining how psychological resources (resilience) and contextual resources (language proficiency, environmental familiarity) jointly influence engagement across a well-defined demand transition. By explicitly examining phase-contingent resource effects, this study evaluates whether SDR mechanisms generalize to multilingual medical education and identifies theoretically grounded, teachable resources that institutions may cultivate to protect engagement during vulnerable clinical transitions.

## 2 Methods

### 2.1 Research design and setting

This study employed a cross-sectional quantitative survey design grounded in a theory-driven explanatory framework to examine the predictors of medical students’ academic engagement. This design was selected to test theory-driven associations and moderation effects specified by the SDR theory within a naturalistic educational setting. Experimental or longitudinal designs were considered but rejected because the primary objective was to evaluate phase-contingent associations across existing training phases rather than inferring causality or temporal change.

The study was conducted at the College of Medicine, Alfaisal University, Riyadh, Saudi Arabia, a private, internationally accredited institution with English-medium instruction and Arabic-dominant clinical practice.

### 2.2 Participants

The target population comprised all enrolled medical students in preclinical years 1–2 and clinical years 3–4 during the 2024 academic year. Recruitment was conducted via institutional email and WhatsApp announcements between June and September 2024.

Sample size determination: *A priori* power analysis using G*Power 3.1 (19) indicated a minimum sample size of *N* = 118 to detect medium effects (*f*^2^ = 0.15) with *α* = 0.05 and power = 0.95 for six predictors. The final analytical sample comprised 422 students, substantially exceeding the minimum power requirements.

Inclusion criteria: Current enrollment in the medical program and ability to complete English-language instruments.

Exclusion criteria: Incomplete responses to the primary outcome (academic engagement) or primary predictors (resilience and language proficiency).

The survey was distributed to 1,200 students, yielding 457 responses (38.1% response rate). After excluding 35 cases with missing data on primary variables (see Section 2.5), the final analytical sample was *N* = 422 (92.3% retention).

### 2.3 Measures

All instruments were selected based on their established psychometric validity in international and medical student populations. Internal consistency was assessed in the present study.

Academic engagement was measured using the Utrecht Work Engagement Scale for Students (UWES-9S) (20), a 9-item instrument assessing vigor (four items), dedication (three items), and absorption (two items), rated on a 0–6 Likert scale. Total engagement was calculated as the mean of all items (*α* = 0.82 in this sample).

Psychological resilience was assessed using the Brief Resilience Scale (BRS) (5), a 6-item unidimensional measure rated on a 1–5 scale. The BRS was selected over longer instruments to minimize respondent burden while retaining robust psychometric performance (*α* = 0.79).

Social support was measured using the Duke Social Support Index (DSSI) (21), a 10-item instrument assessing functional emotional support and social integration. Total scores range from 10 to 30 (*α* = 0.85).

Demographic and contextual variables included self-reported gender (male/female), nationality (Saudi/non-Saudi), training phase (preclinical vs. clinical), prior residence in Saudi Arabia (≥3 years vs. <3 years), Arabic language proficiency (conversational/fluent vs. basic/not proficient), and age (continuous). Note that prior residence was highly unbalanced (92.3% ≥ 3 years), which may limit the precision of estimates for this variable.

### 2.4 Procedures

After providing electronic informed consent, participants completed an online survey consisting of the UWES-9S, BRS, DSSI, and demographic items. The survey took approximately 8–10 min to complete.

Pilot testing: Prior to full deployment, the survey was piloted with 30 medical students (balanced by training phase, sex, and nationality) to assess clarity, feasibility, and response burden. No substantive comprehension problems were identified. Minor wording refinements were made to demographic items only. Pilot data were excluded from the analysis.

### 2.5 Statistical analyses

All analyses were conducted using R (version 4.3.1) following regression-based analytical procedures (22) and adhering to STROBE guidelines (23). The analytical approach consisted of four components: (1) variable coding and preparation, (2) primary regression models, (3) secondary moderation and dimensional analyses, and (4) model diagnostics and validation.

#### 2.5.1 Variable preparation and coding

##### 2.5.1.1 Standardization

Three continuous variables were z-standardized (M = 0, SD = 1) prior to analysis: academic engagement (UWES-9S total), psychological resilience (BRS), and social support (DSSI). Z-standardization enables direct comparison of effect sizes across predictors and facilitates interpretation of interaction terms. Both standardized *β* (change in SD units) and unstandardized β (change in original scale units) are reported to aid interpretation.

##### 2.5.1.2 Binary coding

All binary predictors were dummy-coded (0 = reference, 1 = comparison) consistently across models. Reference categories were preclinical phase (vs. clinical), male sex (vs. female), basic/no Arabic proficiency (vs. fluent/conversational), and <3 years Saudi residence (vs. ≥3 years). Positive coefficients indicate higher engagement in the comparison group.

##### 2.5.1.3 Subscale analyses

Engagement subscales (vigor, dedication, absorption) were analyzed on the original 0–6 Likert scale to preserve interpretability, while predictors remained z-standardized.

##### 2.5.1.4 Verification

Coding accuracy was verified by (1) inspecting regression output, (2) cross-checking coefficient signs against bivariate correlations, and (3) independent replication by two investigators. Extended technical specifications are provided in Supplementary methods S1, including full model equations (S1.2), quality control procedures (S1.3), and software implementation details (S1.6).

#### 2.5.2 Missing data

Missing data patterns were evaluated using Little’s MCAR test and independent t-tests comparing completers and non-completers. Because missingness was minimal (7.6%) and completely at random (*χ*^2^ = 18.3, *p* = 0.14), listwise deletion was applied. Sensitivity analyses using multiple imputation (20 imputations; mice package) confirmed robustness of results.

#### 2.5.3 Primary and secondary analyses

##### 2.5.3.1 Confirmatory model

Hierarchical multiple regression examined academic engagement predicted by resilience, training phase, language proficiency, prior residence, social support, and sex. Standardized and unstandardized coefficients, R^2^, adjusted R^2^, F-statistics, and variance inflation factors (VIFs) were reported.

##### 2.5.3.2 A priori moderation analysis

The Resilience × Clinical Phase interaction was tested to evaluate the phase-contingent effects predicted by SDR theory. For this interaction, resilience was z-standardized and clinical phase dummy-coded (0/1); the product term was created from these centered components.

##### 2.5.3.3 Dimensional analyses

The primary model was replicated separately for vigor, dedication, and absorption subscales to assess differential vulnerability across engagement components.

##### 2.5.3.4 Exploratory analyses

Gender × Language Proficiency, Gender × Clinical Phase, and Resilience × Social Support interactions were examined as exploratory analyses with appropriate caution regarding power and interpretation.

Simple slopes were estimated separately for preclinical and clinical students using the interactions package in R version 4.3.2.

#### 2.5.4 Model diagnostics and validation

##### 2.5.4.1 Assumption testing

Regression diagnostics included VIFs for multicollinearity (all <2.0), Breusch–Pagan tests for heteroscedasticity (HC3 robust standard errors applied), normality tests (Shapiro–Wilk, Q–Q plots), linearity assessment (partial regression plots), independence tests (Durbin–Watson), and influential case detection (Cook’s distance). All assumptions were adequately met (details in Supplementary Tables).

##### 2.5.4.2 Cross-validation

Model generalizability was assessed using 10-fold stratified cross-validation (stratified by sex and training phase). Mean test-set R^2^, root mean squared error (RMSE) and mean absolute error (MAE) were calculated to evaluate out-of-sample performance.

##### 2.5.4.3 Statistical significance

All tests were two-tailed with *α* = 0.05.

#### 2.5.5 Sensitivity analysis for prior residence imbalance

Because prior Saudi residence was highly imbalanced (92.3% in the ≥3 years category), we conducted a *post hoc* sensitivity analysis to test robustness of the main findings. The primary confirmatory model was re-estimated after removing the prior residence covariate, while retaining all other predictors and the same estimation approach (HC3 robust standard errors). We compared model fit (R^2^, adjusted R^2^) and standardized coefficients between the full and reduced models.

### 2.6 Ethical considerations

This study was approved by the Alfaisal University Institutional Review Board (IRB-23054; approved May 29, 2024). All participants provided electronic informed consent. Participation was voluntary without incentives, and withdrawal was permitted at any time without penalty.

## 3 Results

### 3.1 Sample characteristics and measurement properties

The survey was distributed to 1,200 medical students, yielding 457 responses (38.1% response rate). After listwise deletion of missing data on primary variables, the final analytical sample comprised 422 students (92.3% retention). Descriptive statistics, scale reliabilities, and sample composition are summarized in Table 1 and Supplementary Table S1.

**Table 1:** Descriptive statistics, reliability, and sample composition (*n* = 422).

Characteristic	N/M	SD/%	Range/95% CI
Sample composition			
Total analytical sample	422	—	92.3% retention
Male	256	60.7%	—
Female	166	39.3%	—
Saudi national	142	33.6%	—
Non-Saudi international	280	66.4%	—
Preclinical phase	279	66.1%	—
Clinical phase	143	33.9%	—
Fluent/Conversational Arabic	306	72.5%	—
Basic/No Arabic proficiency	116	27.5%	—
Prior Saudi Residence ≥3 years	390	92.3%	—
Primary outcome			
UWES-9S Overall (0–6)	4.15	0.84	[4.07, 4.23]; 1.33–6.00; α = 0.82
Vigor	4.36	0.95	[4.27, 4.45]; 1.00–6.00; α = 0.79
Dedication	3.95	1.12	[3.84, 4.06]; 0.00–6.00; α = 0.81
Absorption	4.14	1.18	[4.03, 4.25]; 0.00–6.00; α = 0.74
Main predictor			
BRS Resilience (1–5)	2.59	0.76	[2.51, 2.65]; 1.00–4.83; α = 0.79
Secondary predictor			
DSSI Social Support (10–30)	23.96	5.69	[23.44, 24.48]; 5.00–30.00; α = 0.85

The sample included 256 male (60.7%) and 166 female (39.3%) students. By nationality, 280 students (66.4%) were non-Saudi international students and 142 (33.6%) were Saudi nationals. Preclinical students comprised 66.1% of the sample (*n* = 279), and clinical students comprised 33.9% (*n* = 143). Conversational or fluent Arabic proficiency was reported by 72.5% of participants (*n* = 306), while 27.5% (*n* = 116) reported basic or no proficiency. The mean age was 21.6 years (SD = 2.1; range 18–35).

### 3.2 Data integrity, regression diagnostics

Missing data occurred in 35 cases (7.6%) and were confined to optional demographic variables (Supplementary Table S2). Little’s test indicated that the data were missing completely at random (*χ*^2^ = 18.3, *p* = 0.14), with no differences between completers and excluded cases on engagement, resilience, or social support (all *p* > 0.05). Sensitivity analyses comparing listwise deletion with multiple imputation yielded equivalent estimates across all predictors (Supplementary Table S3).

Regression diagnostics and assumption checks are summarized in Supplementary Table S4. Heteroscedasticity was detected (Breusch–Pagan test: *χ*^2^ = 12.4, *p* = 0.008) and addressed using HC3 robust standard errors. Residual normality (Shapiro–Wilk W = 0.992, *p* = 0.087), linearity (partial regression plots), independence (Durbin–Watson = 1.94), and influential outliers (Cook’s distance max = 0.087) were within acceptable ranges.

Variance inflation factors ranged from 1.26 to 1.84, indicating no multicollinearity concerns (Supplementary Table S5).

Ten-fold cross-validation demonstrated stable out-of-sample performance for the primary confirmatory model (Model 4), with a mean test-set *R*^2^ of 0.596 (SD = 0.128), indicating minimal overfitting (Supplementary Table S6).

### 3.3 Univariate associations

Bivariate associations among all study variables are presented in Supplementary Table S7. Resilience showed the strongest correlation with engagement (*r* = 0.590, *p* < 0.001), followed by prior residence (*r* = 0.456, *p* < 0.001), and Arabic language proficiency (*r* = 0.376, *p* < 0.001). Clinical training phase showed a negligible correlation with engagement (*r* = 0.050, *p* = 0.547). Social support showed a small negative bivariate association (*r* = −0.127, *p* = 0.009).

Group comparisons revealed lower engagement among female students than among male students (d = 0.49, *p* = 0.009), and substantially lower engagement among students with basic/no Arabic proficiency than among fluent speakers (d = 0.52, *p* < 0.001; Table 2).

**Table 2:** Univariate associations; bivariate correlations and group comparisons (*n* = 422).

Association	Variable/Comparison	Statistic	Value	95% CI	*p*	Effect
Zero-order correlations						
	Resilience	r	0.590	[0.525, 0.647]	<0.001***	35.0% var
	Prior Residence (≥3 yrs)	r	0.456	[0.375, 0.529]	<0.001***	20.8% var
	Language (Fluent)	r	0.376	[0.291, 0.457]	<0.001***	14.1% var
	Social Support	r	−0.127	[−0.221, −0.033]	0.009**	1.6% var
	Clinical Phase	r	0.050	[−0.046, 0.145]	0.547	Negligible
Group comparisons (*t*-tests)						
Gender	Female vs. Male	d	0.488	[0.296, 0.680]	0.009**	Small-Medium
Language	Fluent vs. Basic	d	0.52	[0.31, 0.73]	<0.001***	Medium
Resilience	High (M + 1SD) vs. Low (M − 1SD)	d	1.180	[0.942, 1.418]	<0.001***	Large
Intersectional	Female + Basic vs. Male + Fluent	d	0.91	[0.65, 1.17]	<0.001***	Large

***p < 0.001; **p < 0.01.

Hierarchical regression Models 1–3 are presented in Supplementary Table S8. Model 1 (resilience only) explained 34.8% of variance. Model 2 (adding contextual predictors) increased this to 48.3% (Δ*R*^2^ = 0.135, *p* < 0.001). Model 3 (adding social support) yielded no additional variance.

These univariate patterns informed the specification of the primary confirmatory model.

### 3.4 Primary confirmatory model: main effects

The primary confirmatory model (Model 4) examined the independent contributions of resilience, language proficiency, prior residence, social support, sex, and clinical training phase. Full model estimates are presented in Supplementary Table S9.

Model 4 explained 52.8% of engagement variance (*R*^2^ = 0.528, *p* < 0.001). Resilience emerged as the strongest predictor (*β* = 0.418, *f*^2^ = 0.370), followed by female sex (*β* = −0.410, *f*^2^ = 0.356), Arabic language proficiency (*β* = 0.370, *f*^2^ = 0.267), and prior residence (*β* = 0.345, *f*^2^ = 0.252). Social support showed a small positive association (*β* = 0.104, *f*^2^ = 0.023, *p* = 0.010). Clinical training phase showed no main effect on overall engagement (*β* = −0.004, *p* = 0.920). Results are summarized in Table 3, Panel A.

**Table 3:** Primary confirmatory model and secondary analyses; effects on academic engagement (*n* = 422).

Panel A: Model 4 main effects (all predictors)					
Predictor	Standardized *β* [95% CI]	Unstandardized *β* [95% CI]	*p*	*f* ^2^	Effect Size
Resilience (BRS)	0.418 [0.355, 0.481]	0.462 [0.392, 0.532]	<0.001***	0.370	Large
Language proficiency (Fluent/Conversational)	0.370 [0.225, 0.515]	0.696 [0.424, 0.969]	<0.001***	0.267	Medium
Prior residence (≥3 years Saudi Arabia)	0.345 [0.231, 0.459]	1.084 [0.724, 1.439]	<0.001***	0.252	Medium
Sex (Female)	−0.410 [−0.573, −0.247]	−0.703 [−0.982, −0.424]	<0.001***	0.356	Large
Clinical training phase	−0.004 [−0.083, 0.075]	−0.0071 [−0.148, 0.134]	0.920	<0.001	Negligible
Social support (DSSI)	0.104 [0.025, 0.183]	0.0154 [0.0037, 0.0270]	0.010*	0.023	Small

Panel B: Resilience × Clinical phase moderation (Model 5)					
Training phase	*n*	Standardized resilience *β* [95% CI]	Unstandardized resilience *β* [95% CI]	*p*	*f* ^2^
Clinical (High demand)	143	0.630 [0.460, 0.800]	0.696 [0.509, 0.886]	<0.001***	0.167
Preclinical (Low demand)	279	0.291 [0.169, 0.413]	0.321 [0.187, 0.457]	<0.001***	0.061
Interaction effect	—	0.339 [0.221, 0.457]	0.374 [0.244, 0.506]	<0.001***	0.129

Panel C: Social support × gender stratification (Model 4)					
Sex	*n*	Standardized social support *β* [95% CI]	Unstandardized social support *β* [95% CI]	*p*	*f* ^2^
Female	166	0.06 [−0.11, 0.23]	0.00885 [−0.0163, 0.0341]	0.48	0.003
Male	256	0.14 [0.005, 0.275]	0.0207 [0.00074, 0.0408]	0.04*	0.024

Model fit: *R*^2^ = 0.528, 95% CI [0.497, 0.559]; Adjusted *R*^2^ = 0.520; *F* (6,415) = 72.8, *p* < 0.001. Amplification Factor: 0.696/0.321 = 2.17 × stronger effect in the clinical phase. Model fit: *R*^2^ = 0.565, 95% CI [0.535, 0.595]; *F* (7,414) = 73.1, *p* < 0.001; Δ*R*^2^ = 0.037 (*p* < 0.001).

### 3.5 Moderation analysis: resilience × clinical phase

The resilience × clinical phase interaction was statistically significant [*β* = 0.339, 95% CI (0.221, 0.457), *p* < 0.001, *f*^2^ = 0.129]. Simple slope analyses indicated that resilience showed stronger associations with engagement in clinical students [*β* = 0.630, 95% CI (0.460, 0.800), *f*^2^ = 0.167] than in preclinical students [*β* = 0.291, 95% CI (0.169, 0.413), *f*^2^ = 0.061], representing a 2.17-fold amplification.

**Figure 1:**
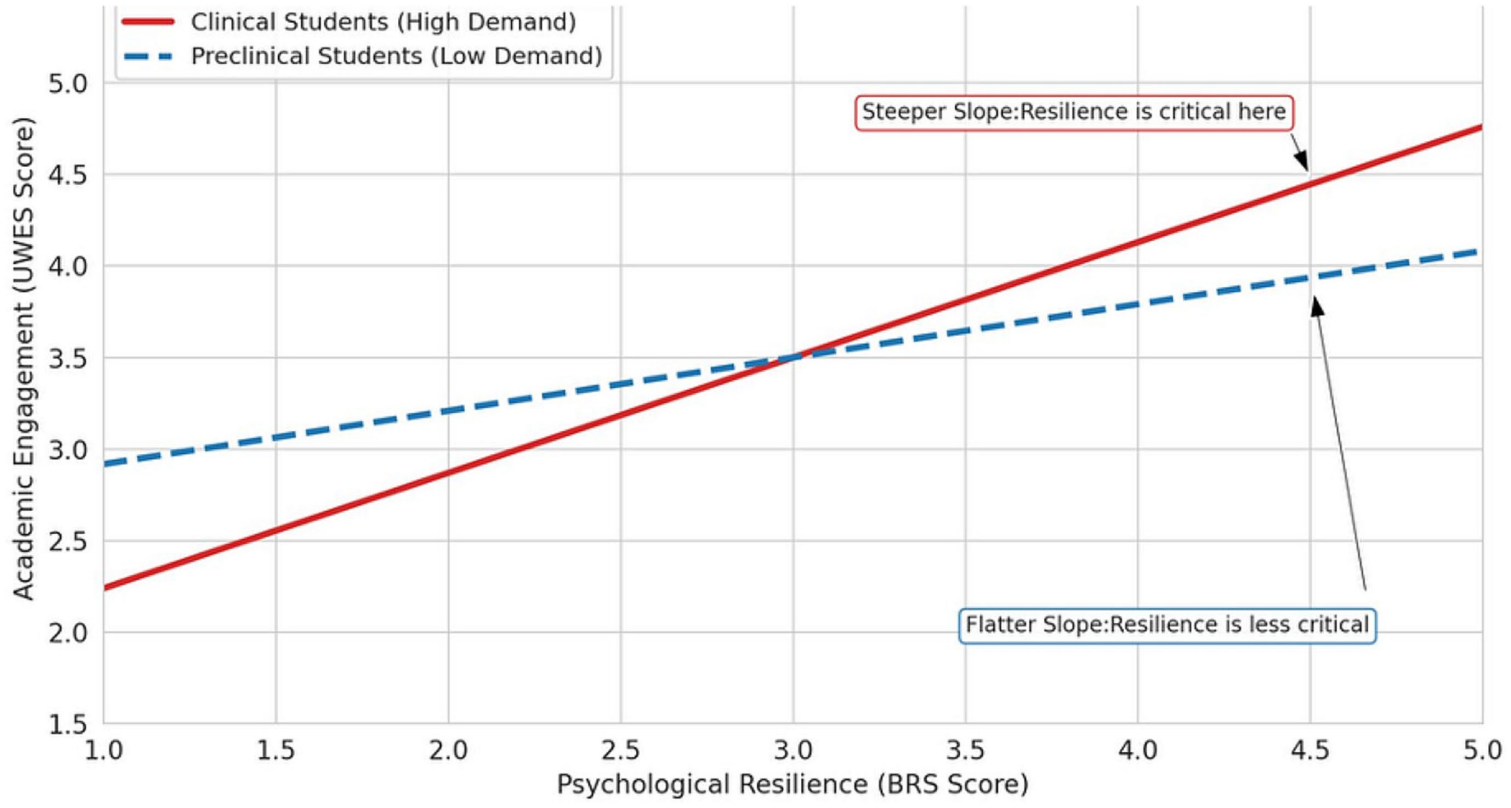
Phase-contingent association between resilience and engagement by training phase.

Including the interaction term increased explained variance from *R*^2^ = 0.528 to *R*^2^ = 0.565 (Δ*R*^2^ = 0.037, *p* < 0.001). Results are summarized in Table 3, Panel B and detailed in Supplementary Table S10. Figure 1 illustrates these phase-specific simple slopes.

Caption: Simple slopes showing the association between resilience and engagement stratified by training phase. In clinical students (Years 3–4), the resilience effect was *β* = 0.630 [95% CI (0.460, 0.800)], compared with *β* = 0.291 [95% CI (0.169, 0.413)] in preclinical students (Years 1–2). The interaction effect was statistically significant [*β* = 0.339, 95% CI (0.221, 0.457), *p* < 0.001, *f*^2^ = 0.129]. Shaded regions represent 95% confidence intervals.

### 3.6 Dimensional analysis: engagement subscales

Dimension-specific regression analyses examining vigor, dedication, and absorption separately are presented in Table 4.

**Table 4:** Subscale regression analyses—engagement dimensions (*n* = 422).

Predictor	Vigor (SD = 0.95)		Dedication (SD = 1.12)		Absorption (SD = 1.18)	
	Std. *β* [95% CI]	Unstd. *β* [95% CI]	Std. *β* [95% CI]	Unstd. *β* [95% CI]	Std. *β* [95% CI]	Unstd. *β* [95% CI]
Resilience (BRS)	0.27*** [0.183, 0.357]	0.338 [0.228, 0.446]	0.55*** [0.464, 0.636]	0.811 [0.684, 0.938]	0.33*** [0.238, 0.422]	0.511 [0.369, 0.653]
Clinical phase	−0.059 [−0.179, 0.061]	−0.119 [−0.360, 0.123]	−0.263*** [−0.399, −0.127]	−0.624 [−0.947, −0.301]	−0.229*** [−0.349, −0.109]	−0.542 [−0.825, −0.259]
Language proficiency (Fluent/Conversational)	0.16* [0.010, 0.310]	0.341 [0.023, 0.661]	0.13** [0.034, 0.226]	0.279 [0.073, 0.483]	0.19*** [0.085, 0.295]	0.407 [0.182, 0.632]
Prior residence (≥3 years)	0.21** [0.094, 0.326]	0.748 [0.335, 1.160]	0.07 [−0.041, 0.181]	0.295 [−0.174, 0.764]	0.15* [0.030, 0.270]	0.665 [0.138, 1.192]
Social support (DSSI)	0.09*** [0.042, 0.138]	0.0150 [0.0070, 0.0230]	0.11*** [0.094, 0.126]	0.0217 [0.0185, 0.0248]	0.07*** [0.023, 0.117]	0.0145 [0.0048, 0.0242]
Sex (Female)	0.19** [0.077, 0.303]	0.370 [0.150, 0.590]	0.06 [−0.057, 0.177]	0.138 [−0.132, 0.408]	−0.02 [−0.131, 0.091]	−0.048 [−0.295, 0.199]
*R* ^2^	0.58		0.62		0.59	
Adjusted *R*^2^	0.576		0.616		0.584	
*F* (df)	96.4* (6,415)		108.6* (6,415)		99.1* (6,415)	

**p < 0.001; **p < 0.01; *p < 0.05. Binary variables coded as 0 = reference category (male, preclinical phase, basic/no Arabic proficiency, <3 years Saudi residence), 1 = comparison category (female, clinical phase, fluent/conversational Arabic proficiency, ≥3 years Saudi residence). Positive standardized coefficients indicate higher engagement scores in the comparison group. All subscale outcomes analyzed on the original 0–6 Likert scale. Continuous predictors (resilience, social support) were z-standardized prior to analysis. HC3 heteroskedasticity-consistent robust standard errors applied to all models. Finally, we conducted exploratory analyses to generate hypotheses for future research.

Resilience showed positive associations with all three engagement dimensions. Clinical training phase was negatively associated with dedication and absorption but not vigor. Language proficiency demonstrated positive associations across all dimensions. Prior residence (≥3 years) showed positive associations with vigor and absorption but not dedication. Social support demonstrated uniformly small positive effects across dimensions.

Gender-stratified regression models are presented in Supplementary Table S11. Among male students, social support showed a small positive association with engagement (*β* = 0.14, *p* = 0.04), whereas among female students the association was not statistically significant (*β* = 0.06, *p* = 0.48).

### 3.7 Exploratory analyses

Exploratory moderation analyses examined Gender × Language Proficiency and Gender × Clinical Phase interactions. Neither interaction reached statistical significance (*p* = 0.087 and *p* = 0.56, respectively).

Power analyses confirmed adequate power (>0.85) for the primary confirmatory model and the resilience × phase moderation analysis, but insufficient power (0.60–0.72) for exploratory interactions (Supplementary Table S12). A comprehensive synthesis of effect sizes across all confirmatory and exploratory analyses is presented in Supplementary Table S13.

### 3.8 Sensitivity analysis

Sensitivity analysis excluding prior residence showed minimal change in model performance and coefficient estimates. The full model explained *R*^2^ = 0.528, and the reduced model (without prior residence) explained *R*^2^ = 0.525 (Δ*R*^2^ = −0.003). Standardized coefficients remained stable, with resilience changing from 0.418 to 0.422, Arabic language proficiency remaining 0.370, sex changing from −0.410 to −0.411, social support changing from 0.104 to 0.099, and clinical phase remaining −0.004. Statistical significance patterns for the primary predictors were unchanged. These findings indicate that the main conclusions are robust to exclusion of the imbalanced prior residence variable (Supplementary Table S14).

## 4 Discussion

### 4.1 Hypothesis evaluation and overview of findings

This study provides theory-consistent, hypothesis-generating evidence supporting the central propositions derived from SDR theory within a multilingual medical education context. Psychological resilience and Arabic language proficiency were independently associated with academic engagement, with resilience showing the largest effect size (*f*^2^ = 0.370). The resilience–engagement association was substantially stronger during the clinical phase than during the preclinical phase (*β* = 0.630 vs. *β* = 0.291), consistent with phase-contingent amplification of resource effects. Language proficiency and prior environmental familiarity showed independent associations with engagement beyond resilience, suggesting that contextual and linguistic resources may operate alongside psychological resources. Social support showed small positive associations across all engagement dimensions (*β* = 0.07–0.11), with minimal dimension-specific variation.

Dimensional analyses revealed that clinical training phase differentially affected engagement subscales: dedication and absorption declined while vigor remained stable. Female students reported lower overall engagement but demonstrated non-uniform patterns across subscales, with higher vigor but non-significant differences in dedication and absorption when controlling for other predictors.

Together, these findings suggest that engagement in multilingual medical education is associated with interacting psychological, contextual, and linguistic factors, with evidence consistent with but not establishing variation in resource effectiveness by demand intensity.

### 4.2 Primary finding: phase-contingent resilience effects

The central finding of this study was that resilience–engagement associations differed substantially by training phase. Among clinical students, resilience showed a strong association with engagement [*β* = 0.630, 95% CI (0.460, 0.800)]; among preclinical students, the association was approximately half as strong [*β* = 0.291, 95% CI (0.169, 0.413)]. The resilience × clinical phase interaction was statistically significant [*β* = 0.339, *p* < 0.001, *f*^2^ = 0.129], with the interaction term explaining an additional 3.7% of variance beyond main effects alone.

This phase-contingent pattern is consistent with SDR theory’s prediction that psychological resources become more consequential as situational demands intensify (14, 15, 24, 25). Clinical training introduces compounded cognitive, emotional, and performance demands compared with preclinical training (26, 27), potentially creating conditions in which resilience, defined as the capacity to bounce back from adversity (5), functions as a more strongly associated correlate of engagement.

However, three considerations require cautious interpretation. First, training phase is an indirect proxy for demand rather than a direct measure of experienced workload, stress, or performance pressure. While clinical training is consistently associated with higher demands (26, 27), we did not measure demands directly using validated instruments. Second, the cross-sectional design cannot distinguish demand-related amplification from alternative mechanisms, including selection effects, developmental maturation, or unmeasured confounding. Third, students self-selected into this survey (38.1% response rate), potentially introducing selection bias if more resilient students were differentially likely to participate.

Despite these limitations, the observed pattern provides hypothesis-generating evidence supporting the proposition that psychological resources may become more consequential as situational demands intensify, a core tenet of SDR and JD-R frameworks (14, 15, 24).

### 4.3 Contextual resources: language proficiency and environmental familiarity

Arabic language proficiency emerged as a substantial predictor of engagement (*β* = 0.370, *f*^2^ = 0.267), demonstrating consistent positive associations across all engagement dimensions. This broad-spectrum pattern aligns with translanguaging theory, which conceptualizes linguistic competence as a resource facilitating meaning-making and communication in complex clinical contexts (12, 13, 28).

In multilingual medical education settings where instruction occurs in English, but patient care predominantly involves Arabic, linguistic competence may be associated with lower cognitive load, more effective clinical communication, and stronger professional identity development (8–11, 16–18). These findings challenge deficit-based framings of multilingualism as an obstacle to learning (29), instead supporting conceptualization of language competence as a learnable, engagement-relevant resource in clinical education (30).

Prior Saudi residence (≥3 years) showed a positive association with overall engagement but selective subscale effects. This suggests that environmental familiarity may reduce cognitive load related to navigating cultural norms and healthcare system structures, preserving energy and facilitating immersion without necessarily increasing perceived meaningfulness.

Hierarchical regression demonstrated that contextual resources substantially increased explained variance beyond resilience alone, reinforcing that engagement reflects interaction between personal and contextual resources rather than individual traits in isolation.

### 4.4 Psychological resources: social support

Social support showed small positive associations with all engagement dimensions (vigor *β* = 0.09, dedication *β* = 0.11, absorption *β* = 0.07, all *p* < 0.001), with minimal variation across subscales. This uniformly modest pattern is consistent with prior research suggesting that peer and faculty relationships support student well-being and academic outcomes (6, 27), though effect sizes in our study were substantially smaller than those typically reported for proximal psychological resources like resilience.

Several factors may explain the limited associations. First, our 10-item generic social support scale (DSSI-10) (21) may not adequately capture domain-specific support most relevant to medical student engagement, such as faculty mentorship quality, peer learning collaboration, or clinical preceptor relationships. Generic measures may be less sensitive to the specific forms of support that buffer against academic demands or promote professional development. Second, the bivariate correlation between social support and overall engagement was negative (*r* = −0.127, *p* = 0.009), becoming positive only after controlling for sex, suggesting suppression effects related to gender differences in social support networks (31). Third, post-hoc power analysis revealed insufficient statistical power (0.189) for detecting social support effects, well below the conventional 0.80 threshold, limiting interpretive confidence.

From a theoretical perspective, social support may operate more distally than proximal psychological resources like resilience within the SDR framework (14, 15). While resilience represents an immediately deployable internal capacity theorized to buffer against demands, social support requires activation through help-seeking behavior and may take longer to translate into engagement benefits. This distal positioning could explain why social support effects appear smaller and less differentiated across engagement dimensions.

Gender-stratified analyses (Table 3, Panel C; Supplementary Table S11) revealed that social support associations with engagement were statistically significant among male students (*β* = 0.14, *p* = 0.04) but not among female students (*β* = 0.06, *p* = 0.48), though both effects were small (*f*^2^ = 0.024 and 0.003, respectively). The mechanisms underlying this pattern remain unclear and warrant investigation using domain-specific support measures and qualitative methods examining how male and female students perceive and utilize different forms of support.

Future research should employ validated, domain-specific social support measures (e.g., faculty mentorship scales, peer learning support inventories, clinical supervisor relationship quality) to clarify whether stronger dimension-specific effects emerge when support is measured with greater precision. Longitudinal designs tracking changes in social support networks during the clinical transition would illuminate whether support becomes more consequential as demands intensify.

Social support showed small positive associations with engagement dimensions. This pattern is broadly consistent with literature indicating that supportive peer and faculty relationships promote well-being and academic functioning (6, 27), although effect sizes were modest. Several explanations merit consideration. The DSSI-10 (21) is a generic support measure and may not capture domain-specific academic support. The negative bivariate association that became positive after adjustment suggests possible suppression effects (31). Power for detecting small effects was limited. Within SDR frameworks, social support may operate as a more distal resource relative to proximal psychological capacities like resilience (14, 15). Its benefits may depend on activation processes and contextual contingencies.

### 4.5 Dimensional patterns across engagement subscales

Clinical training phase showed minimal association with overall engagement (*β* = −0.004, *p* = 0.920) but demonstrated clear dimension-specific patterns when subscales were examined separately. Clinical students reported significantly lower dedication (*β* = −0.263, *p* < 0.001) and absorption (*β* = −0.229, *p* < 0.001) but showed no difference in vigor (*β* = −0.059, *p* = 0.305) compared with preclinical students, after controlling for all other predictors.

This pattern suggests that clinical training may be associated with qualitative differences in engagement rather than uniform reductions. The preservation of vigor (energy, mental resilience, persistence) alongside declines in dedication (enthusiasm, inspiration, pride) and absorption (concentration, immersion, time distortion) may reflect adaptation to sustained high-demand environments where maintaining energy becomes prioritized over experiencing flow states or deriving meaning from tasks (1, 2). Alternatively, it could indicate that clinical training introduces competing demands (patient care responsibilities, documentation burden, interpersonal complexity) that fragment attention and reduce opportunities for deep immersion, even as students maintain adequate energy levels through effective resource mobilization (32).

The resilience associations also varied by dimension, with the strongest effect observed for dedication (*β* = 0.55) compared with absorption (*β* = 0.33) and vigor (*β* = 0.27). This suggests that resilience may be particularly strongly associated with maintaining a sense of meaning. These elements are central to preventing burnout and sustaining long-term career engagement (26, 33).

These findings underscore that reliance on aggregate engagement scores may obscure meaningful domain-specific effects (32). From a measurement perspective, the UWES-9S structure with equal item representation (vigor: 3 items, dedication: 3 items, absorption: 3 items) may still complicate interpretation when only total scores are analyzed (20). Dimension-specific analyses appear necessary to capture how engagement is reshaped under heightened demand conditions and how different resources selectively support specific engagement facets.

Clinical training phase showed dimension-specific effects. Dedication and absorption declined, while vigor remained stable. This pattern is compatible with engagement theory distinguishing vigor, dedication, and absorption as related but separable constructs (1, 2, 20). Preservation of vigor alongside reduced dedication/absorption may reflect adaptation to sustained demands, consistent with burnout and occupational strain literature (32). Resilience effects were strongest for dedication, suggesting particular relevance for sustaining meaning and commitment under pressure, elements closely linked to burnout prevention (26, 33). These findings reinforce that aggregate engagement scores may obscure domain-specific effects (32).

### 4.6 Gender differences in engagement: descriptive patterns requiring further investigation

Female students reported lower overall engagement (*β* = −0.410, *p* < 0.001, *f*^2^ = 0.356) in the primary multivariate model controlling for all predictors. However, dimension-specific analyses revealed non-uniform patterns: female students demonstrated higher vigor (*β* = +0.19, *p* = 0.001) but non-significant differences in dedication (*β* = +0.06, *p* = 0.42) and absorption (*β* = −0.02, *p* = 0.78). The discrepancy between the large negative main effect on overall engagement and the mixed subscale patterns (positive vigor, null dedication/absorption) requires cautious interpretation.

Several methodological factors complicate interpretation. First, overall engagement is calculated as the mean of nine items distributed across three subscales, and composite scoring may mask dimension-specific behavior. Second, gender correlated with other predictors, particularly social support (*r* = −0.178, *p* < 0.01), suggesting potential suppression effects wherein the apparent main effect emerges primarily through covariate adjustment rather than representing a direct bivariate association (31). Third, the cross-sectional design and reliance on self-report measurement introduce possibilities for response-style differences or measurement artifacts that cannot be ruled out.

Critically, our study design does not permit causal interpretation of these patterns. We did not measure factors that could plausibly explain gender differences, such as educational climate perceptions, experiences of discrimination or microaggressions, access to mentorship networks, clinical rotation assignment patterns, peer interaction quality, or psychosocial stressors outside of training. Without such measures, the mechanisms underlying these patterns remain unclear.

These findings should be interpreted as descriptive patterns warranting further investigation rather than as established gender effects with known causes. Future research should employ qualitative methods to illuminate whether and how male and female students experience engagement differently, longitudinal designs to examine whether gender differences emerge at specific training transitions, multi-method assessment combining self-report with objective indicators (e.g., clinical participation, academic performance), and intersectional analyses examining how gender intersects with other identities (nationality, language proficiency, socioeconomic status) (34).

### 4.7 Alternative explanations and causal limitations

The observed phase-contingent resilience association is consistent with SDR theory’s demand-moderation mechanism but does not definitively establish this mechanism. Several alternative explanations warrant detailed consideration, all of which arise from the study’s cross-sectional design and indirect operationalization of demand through training phase.

#### 4.7.1 Selection and survivor effects

Students entering clinical training may differ systematically from those in preclinical years due to selective attrition. If less resilient students are more likely to struggle academically, experience burnout, or leave the program during preclinical years, the clinical cohort would represent a selected sample with higher baseline resilience or greater capacity to leverage psychological resources effectively. This would inflate resilience–engagement associations in the clinical group independently of any demand-related amplification.

Several mechanisms could produce this pattern. First, academic performance requirements may disproportionately filter out students with lower resilience during preclinical years, when summative examinations determine progression. Students who fail or repeat courses may subsequently withdraw, creating an increasingly resilient clinical cohort. Second, psychological distress and burnout, both inversely associated with resilience (26, 33) may drive voluntary attrition during preclinical training. Third, financial pressures may interact with resilience; students with limited financial resources and low resilience may be more likely to withdraw when facing academic difficulties, whereas resilient students may persist and seek support.

Our data cannot distinguish these selection mechanisms from demand-related amplification because we lack information on: (a) attrition rates across training phases; (b) baseline characteristics (resilience, engagement, academic performance) of students who withdrew versus those who remained; (c) reasons for withdrawal (academic failure, psychological distress, financial constraints, career reorientation); and (d) whether resilience measured at matriculation predicts persistence through preclinical training. Without prospective tracking of entire cohorts from entry through graduation, selection effects remain a plausible alternative explanation.

#### 4.7.2 Developmental maturation and life experience

Clinical students are typically 2–3 years older than preclinical students (mean age in our sample preclinical 20.8 years, clinical 23.1 years) and may have accumulated developmental advantages unrelated to training demands. Several maturation-related mechanisms could strengthen resilience effects independently of demand intensity:

Neurobiological maturation: Prefrontal cortex development continues through the mid-20s, with adolescent brain maturation influenced by biological and environmental factors that can enhance executive functions, including emotion regulation, impulse control, and strategic planning (35). These capacities may enable more effective resource mobilization and adaptive coping, strengthening the resilience–engagement link even in the absence of demand changes.
Improved metacognitive awareness: With accumulated academic experience, clinical students may have developed stronger self-monitoring skills, enabling earlier recognition of stress signals and more effective deployment of resilience strategies.
Identity consolidation: Older students may have achieved greater clarity regarding career goals, personal values, and professional identity, factors that could interact with resilience to sustain engagement (36).
Life experience accumulation: Clinical students have experienced more academic transitions, interpersonal challenges, and personal setbacks, potentially providing a broader repertoire of coping strategies and stress inoculation.

The cross-sectional design conflates age, training phase, and demand, making it impossible to isolate which factor drives the observed interaction. Longitudinal studies tracking students before, during, and after the clinical transition while controlling for age effects are needed to separate developmental maturation from demand-related mechanisms.

#### 4.7.3 Unmeasured confounding

Clinical and preclinical students may differ on many unmeasured variables that interact with resilience to produce the observed phase-contingent pattern:

*Motivation and career commitment*: Students entering clinical training may be more intrinsically motivated or committed to medical careers than those still in preclinical years. Self-determination, expectancy-value, and achievement goal frameworks all suggest motivation influences how students engage with learning demands and deploy psychological resources (36). Higher motivation could interact with resilience, creating stronger engagement associations in clinical students independently of demand levels.
*Social support network quality*: Clinical students may have developed stronger peer networks, closer faculty mentorship, or more supportive personal relationships. These unmeasured social resources could moderate resilience–engagement associations.
*Financial stability*: Students who persist into clinical training may have resolved financial concerns through loans, family support, or scholarships, whereas preclinical students may be more financially stressed.
*Living arrangements and personal circumstances*: Clinical students may be more likely to have stable housing or fewer unresolved personal disruptions competing for attention.
*Previous clinical exposure*: Some students may have prior healthcare work experience, volunteering, or shadowing, potentially interacting with both training phase and resilience.

Our models controlled for measured covariates (sex, language proficiency, prior residence, social support) but could not account for these unmeasured factors. Comprehensive assessment of potential confounders would require extensive baseline data collection and careful covariate selection based on directed acyclic graph analysis.

#### 4.7.4 Measurement timing and within-phase heterogeneity

The cross-sectional design introduces temporal ambiguity that precludes causal interpretation. We cannot distinguish whether stronger resilience–engagement associations in clinical students reflect: (a) acute demand-related amplification during clinical training; (b) stable between-phase differences existing before transition; or (c) reverse causality, wherein high engagement reinforces resilience over time.

Additionally, students within each training phase likely experience substantial heterogeneity in demand levels that our phase-based operationalization obscures:

*Clinical phase heterogeneity*: Clinical students rotate through diverse specialties (e.g., surgery vs. psychiatry; emergency medicine vs. Obstetrics and Gynecology) with markedly different workload intensities, emotional demands, and learning environments. Clinical sites also vary (academic centers vs. community hospitals vs. rural clinics), and patient acuity, supervision quality, and institutional culture further differentiate demand exposure.
*Preclinical phase heterogeneity*: Preclinical students also face fluctuating demands depending on curricular timing, course load, and personal circumstances.

Averaging resilience–engagement associations across these heterogeneous within-phase experiences may mask important dose–response relationships.

#### 4.7.5 Reverse causality

While we conceptualize resilience as a resource that promotes engagement, engagement may also reinforce resilience over time. Students with high engagement may develop stronger confidence, self-efficacy, and professional identity, all of which could strengthen resilience. This bidirectional relationship cannot be disentangled with cross-sectional data.

#### 4.7.6 Implications for causal inference

These alternative explanations do not invalidate the observed phase-contingent pattern, but they indicate that this study is hypothesis-generating rather than definitive for SDR’s demand-moderation mechanism. The findings are consistent with SDR predictions but could also align with: (a) survivor-bias processes producing progressively high-functioning cohorts; (b) developmental stage processes in which maturation improves resource utilization (35, 36); (c) resource-accumulation frameworks; or (d) dose–response relationships obscured by phase-level categorization.

To test whether demand intensity moderates resource–engagement associations, future studies should:

Include direct demand measurement using validated multidimensional instruments (e.g., cognitive workload scales, workload subscales, weekly clinical hours, psychological demand measures, emotional labor measures).

- Employ longitudinal within-person designs tracking students from matriculation through clinical completion with repeated measurement of resilience, engagement, and demands.
- Test moderation within training phases by comparing high-demand periods (exam weeks, intensive rotations) against lower-demand periods (breaks, electives).
- Evaluate selection processes by assessing initial resilience and engagement levels, monitoring dropout rates, and analyzing the differences between those who remain and those who leave.
- Use real-time repeated assessments to capture day-to-day variation in demands, resource mobilization, and engagement.
- Implement quasi-experimental designs that evaluate engagement changes when curricular demand is altered (e.g., protected time, workload policies, schedule restructuring).

Such designs would provide stronger causal evidence regarding whether, when, and how demand intensity moderates resource–engagement associations in medical education.

### 4.8 Positioning within the literature

These findings align with prior SDR and JD-R research demonstrating that resources exert stronger effects under elevated-demand conditions (14, 15, 24, 25). Although much of this literature originates from occupational settings, the present findings extend these mechanisms to medical education, where clinical training introduces compounded cognitive, emotional, and linguistic demands (26, 27, 37). Recent applications of SDR to medical student populations have similarly documented associations between study-related resources (academic self-efficacy, social support, autonomy) and engagement outcomes (25), though few have examined phase-contingent effects or multilingual contexts.

The observed language proficiency effects contribute to emerging literature challenging deficit-based framings of multilingualism in medical education (8, 9, 29). Rather than conceptualizing language diversity solely as a barrier to learning, these findings support translanguaging perspectives emphasizing flexible language use as a means of enhancing meaning-making, participation, and professional communication in complex clinical environments (12, 13, 28). Studies from Arabian Gulf contexts similarly document that language proficiency facilitates clinical learning, patient communication, and professional confidence among medical trainees (10, 11, 16–18), though few have quantified these associations using validated engagement measures or examined interactions with other resources.

The dimension-specific engagement patterns observed in this study align with prior research suggesting that vigor, dedication, and absorption may respond differentially to environmental demands and personal resources (1, 2, 20, 32). The preservation of vigor alongside declines in dedication and absorption during clinical training echoes patterns observed in high-demand occupational settings, where individuals prioritize energy maintenance over experiential engagement when faced with sustained stressors (32).

### 4.9 Cultural and educational context: gulf region considerations

The Gulf region presents a distinctive cultural and educational context that likely influenced the patterns observed in this study. Understanding these contextual factors is essential for interpreting findings and assessing their generalizability to other settings.

#### 4.9.1 Linguistic landscape and English-medium instruction

Saudi Arabia, like other Gulf Cooperation Council nations, has rapidly expanded medical education capacity over the past two decades, retaining strong use of English-medium instruction despite Arabic being the primary language for many students and patients (8, 9, 16). This linguistic model creates demands distinct from settings where instructional and clinical languages align.

The English-medium model in Gulf medical schools reflects policy and accreditation pressures and aspirations for international clinical readiness (8). However, this model places students in ongoing linguistic translation: learning complex scientific content in English while conducting much patient communication in Arabic. Prior Gulf-context research indicates this mismatch can raise cognitive load and challenge clinical communication (9–11, 16, 17).

Our finding that Arabic language proficiency was a strong, independent predictor of engagement (*β* = 0.370, *f*^2^ = 0.267) likely reflects these demands. In Anglophone schools, language proficiency is often a baseline requirement rather than a major source of variation. In Gulf settings, language can function as a dynamic resource deployed across instructional and clinical contexts. Students with stronger Arabic proficiency may encounter lower communication-related load and greater confidence in patient interaction and clinical reasoning, supporting engagement (10, 30).

The dimension-specific pattern, with language proficiency associated across vigor, dedication, and absorption, suggests linguistic competence functions as a broad resource. This challenges deficit-oriented framings that position multilingualism mainly as a barrier (8, 29).

#### 4.9.2 Cultural context: collectivism, gender norms, and social hierarchy

Gulf societies are shaped by collectivist orientations, family obligations, hierarchical social structure, and gender-differentiated role expectations (27, 38). These contextual features may influence how students experience and respond to medical training demands.

Collectivism and social support: In collectivist settings, group harmony and interdependence may shape help-seeking and support activation (38). The modest social support effects in our data (*β* = 0.09–0.11) are smaller than some Western reports (39), potentially due to measurement limitations or context-specific support pathways (e.g., family-centered support not fully captured by generic instruments).

Gender norms and training: Traditional expectations in Gulf and South Asian contexts may influence professional trajectories and social responsibilities (38, 40). Female physicians in Saudi Arabia increasingly participate across specialties but may still navigate distinct expectations that can affect training experience (40). These contextual factors may relate to observed engagement differences, though causal interpretation is not possible in this design.

Hierarchical clinical learning environments: Gulf healthcare systems often feature strong authority gradients (37, 41). Such structures may reduce learner autonomy and shape how resources such as resilience translate into engagement under supervisory constraints.

#### 4.9.3 Educational system characteristics

The study institution, like many Gulf schools, includes a large international student segment (66.4% non-Saudi), producing diverse linguistic and educational backgrounds. Prior residence in Saudi Arabia (≥3 years) may reflect adaptation to local educational and healthcare norms beyond language alone. As a private institution, contextual features may differ from public settings in support structures, resources, and selectivity.

Gulf healthcare systems have undergone rapid modernization while retaining local organizational features (41, 42). Students learning in these environments may face combined clinical, linguistic, and cultural demands, which can increase the relevance of both psychological and contextual resources.

#### 4.9.4 Implications for interpretation and generalizability

First, the language effects seen here may be less pronounced in settings where instructional and clinical languages are aligned. Second, social support effect sizes may vary by culture, hierarchy, and measurement approach. Third, gender patterns require cautious interpretation in view of unmeasured structural variables. Fourth, phase-contingent resilience effects may be amplified by multilingual and hierarchical demands in this setting. Finally, these findings support context-sensitive medical education research rather than assuming identical mechanisms across all systems.

### 4.10 Contributions of this study

#### 4.10.1 Theoretical contribution

This study provides hypothesis-generating evidence consistent with the phase-contingent resource mechanism of SDR theory in medical education, extending applicability to multilingual clinical training contexts (14, 15, 24, 25). By documenting stronger resilience–engagement associations during clinical training, the findings support the proposition that psychological resources become more consequential as demands intensify, while still allowing alternative explanations given the cross-sectional design.

#### 4.10.2 Contextual contribution

This study addresses a gap by examining engagement where instructional language (English) differs from patient-care language (Arabic). Findings indicate that linguistic competence can operate as an engagement-relevant resource alongside resilience and environmental familiarity (8–11, 16–18).

#### 4.10.3 Methodological contribution

By contrasting preclinical and clinical phases and conducting subscale-level analyses, this study shows that aggregate engagement scores may obscure meaningful domain-specific effects (32). It also highlights unresolved ambiguity in cross-sectional studies, including the challenge of separating demand-related amplification from survivor effects and maturation processes (26, 33).

### 4.11 Future research directions

To test the mechanisms suggested by these findings and address limitations, future studies should prioritize the following:

*Longitudinal within-person designs*: Track students prospectively from matriculation through clinical training with repeated measures of resilience, engagement, and direct demand indicators (workload, stress, clinical hours).
*Direct demand measurement*: Use validated demand instruments rather than training phase as a proxy, and test dose–response relationships.
*Mechanistic studies*: Examine cognitive load, language-processing efficiency, and help-seeking as potential mediators between resources and engagement.
*Domain-specific social support measurement*: Use validated tools for faculty mentorship, peer learning support, and clinical supervisor relationship quality.
*Qualitative investigations*: Explore lived experiences of engagement across phases, language demands, and gendered educational experiences.
*Multi-site replication*: Replicate across linguistic and institutional contexts to assess transportability.
*Intervention studies*: Test whether resilience-building interventions have stronger effects during clinical vs. preclinical training, as predicted by SDR demand-moderation logic (43).

### 4.12 Strengths and limitations

#### 4.12.1 Strengths

This study used validated instruments (UWES-9S, BRS, DSSI-10) (5, 20, 21), tested theory-derived hypotheses with appropriate analyses (hierarchical regression, moderation), and achieved adequate power for primary analyses (>0.85 for main effects and the resilience × clinical phase interaction). The sample included representation across nationality, training phase, and language-proficiency strata, supporting contextual analyses uncommon in engagement research. Data quality was strong, with limited missingness (7.6% listwise deletion), acceptable reliability (*α* = 0.74–0.85), and no major regression-assumption violations after robust standard errors.

#### 4.12.2 Limitations

The single-institution private-school setting limits generalizability. The 38.1% response rate introduces possible non-response bias; respondent–nonrespondent comparisons were not available. Cross-sectional self-report measurement may inflate associations through common method variance and cannot establish causality. Binary operationalization of language proficiency and prior residence may miss nonlinear relationships. Power for small social-support effects was limited (post-hoc achieved power = 0.189). Training phase was an indirect demand proxy rather than a direct workload/stress measure. Although clinical training is commonly associated with higher demands (26, 27), unmeasured within-phase heterogeneity and alternative explanations (selection/survivor effects (33), developmental maturation (35, 36), unmeasured confounding (36)) remain possible. Future work with direct demand measures and longitudinal designs is needed to test whether phase-contingent patterns reflect genuine demand-moderation processes predicted by SDR theory.

## 5 Conclusion

This cross-sectional study found that the association between resilience and academic engagement was stronger in clinical students than in preclinical students (*β* = 0.630 vs. *β* = 0.291; Δ*R*^2^ = 0.037). Arabic language proficiency and prior Saudi residence were also independently associated with engagement and resilience. These findings are consistent with the Study Demands–Resources framework in a multilingual medical education setting where instructional and clinical languages differ.

These results are associative and should not be interpreted as causal. Training phase was used as an indirect demand marker, and alternative explanations (selection effects, maturation, and unmeasured confounding) remain possible. Longitudinal within-person studies with direct demand measures are needed to test these mechanisms and to assess transferability across settings. If replicated, the findings may help guide timing of resilience-support strategies and language-support approaches in multilingual programs.

## Data Availability

The original contributions presented in the study are included in the article/Supplementary material, further inquiries can be directed to the corresponding author.
