# Correction: Phase-contingent resilience effects in multilingual medical students: a cross-sectional examination of student demands-resources theory

**DOI:** 10.3389/fmed.2026.1852632

**Published:** 2026-05-12

**Authors:** Sadia Qazi, Siyaan Yasser Qureshi, Eshal Atif, Muhammad Atif Mazhar, Ismail Mohammed, Mahdi Ameer, Akef Obeidat

**Affiliations:** 1 Department of Anatomy, College of Medicine, Alfaisal University, Riyadh, Saudi Arabia; 2 College of Medicine, Alfaisal University, Riyadh, Saudi Arabia

**Keywords:** clinical training, cross-sectional study, language proficiency, multilingual medical education, psychological resilience, student engagement

An incorrect **Funding** statement was provided. The correct **Funding** statement reads:

The author(s) declared that financial support was received for this work and/or its publication. The APC is covered by Alfaisal University.

The **Ethics statement** was erroneously given as:

The studies involving humans were approved by Institutional Review Board, Alfaisal University. The studies were conducted in accordance with the local legislation and institutional requirements. The participants provided their written informed consent to participate in this study.

The correct **Ethics statement** is:

This study was approved by the Alfaisal University Institutional Review Board (IRB20312; approved May 29, 2024). All participants provided electronic informed consent. Participation was voluntary without incentives, and withdrawal was permitted at any time without penalty.

The original version of this article has been updated.

