# Supplementary material for: Phase-contingent resilience effects in multilingual medical students: a cross-sectional examination of student demands-resources theory
Source: Front Med (Lausanne). 2026 Mar 16;13:1775910. doi: 10.3389/fmed.2026.1775910 (PMC13033520; doi:10.3389/fmed.2026.1775910)
Supplement: Supplementary file 1 [file Table_1.DOCX]

**Phase-contingent resilience effects in multilingual medical students: a cross-sectional examination of Student Demands-Resources theory**

**SUPPLEMENTARY METHODS S1: Variable Coding and Model Specifications**

**S1.1 Variable Standardization**

**Continuous variables** were z-standardized (M = 0, SD = 1): UWES-9S engagement (original M = 4.15, SD = 0.84), BRS resilience (M = 2.59, SD = 0.76), DSSI social support (M = 23.96, SD = 5.69). **Subscale analyses** (Table 4) used original 0–6 scales to preserve clinical interpretability.

**Binary variables** were dummy-coded (0/1) with reference categories: male (n = 256, 60.7%), preclinical phase (n = 279, 66.1%), basic Arabic (n = 116, 27.5%), <3 years Saudi residence (n = 32, 7.6%). Positive coefficients indicate the coded group (1) scored higher than the reference group (0).

**S1.2 Model Equations**

**Model 4 (Primary):**

Engagement_z = β₀ + β₁(Resilience_z) + β₂(Clinical₀/₁) + β₃(Language₀/₁) + β₄(Prior Res₀/₁) + β₅(Social Support_z) + β₆(Female₀/₁) + ε

Where subscript "z" indicates z-standardized variables (M=0, SD=1), subscript "0/1" indicates dummy-coded binary variables, and ε represents the error term with HC3 robust standard errors applied.

**Model 5 (Interaction):**

Adds interaction term: β₇(Resilience_z × Clinical₀/₁)

Simple slopes analysis:

- Preclinical students (Phase = 0): β = 0.291, 95% CI [0.169, 0.413]
- Clinical students (Phase = 1): β = 0.630, 95% CI [0.460, 0.800]
- Amplification factor: 2.16× (clinical effect is 2.16 times stronger than preclinical)

**Subscale Models (Table 4):**

Identical to Model 4 equation, except the outcome variable uses the original 0–6 Likert scale (not z-standardized) to preserve clinical interpretability:

Subscale₀₋₆ = β₀ + β₁(Resilience_z) + β₂(Clinical₀/₁) + β₃(Language₀/₁) + β₄(Prior Res₀/₁) + β₅(Social Support_z) + β₆(Female₀/₁) + ε

Where Subscale ∈ {Vigor, Dedication, Absorption}.

**S1.3 Quality Control**

**Verification procedures:**

1. **Standardization:** Confirmed all z-scored variables had M ≈ 0.00, SD ≈ 1.00
2. **Multicollinearity:** All VIF < 2.0 (range 1.26–1.84)
3. **Coefficient consistency:** All standardized β signs matched bivariate correlations except social support (expected suppression effect after controlling sex)
4. **Independent replication:** Two analysts re-ran all models; coefficients matched to 3 decimals
5. **Heteroscedasticity:** Breusch-Pagan test detected heteroscedasticity (χ² = 12.4, p = 0.008); HC3 robust standard errors applied

**Software:** R version 4.3.1; packages: lm(), car::vif(), sandwich::vcovHC() (HC3), interactions::sim_slopes().

**S1.4 Methodological Rationale**

**Dummy coding (vs. effects coding):** Chosen for interpretability with unbalanced groups (e.g., 92% ≥3 years residence).
**HC3 robust SEs:** Conservative finite-sample correction for detected heteroscedasticity.
**Z-standardization:** Enables effect size comparison across predictors on different scales.

**S1.5 Transparency Statement**

**Pre-registration:** Not pre-registered. Resilience × phase interaction specified a priori; other interactions exploratory.
**Data/code availability:** De-identified dataset and complete R scripts available from corresponding author upon reasonable request, subject to IRB approval.

**SUPPLEMENTARY TABLE S1. Descriptive Statistics, Reliability, and Sample Composition (N = 422)**

| **Characteristic** | **N / M** | **SD / %** | **Range / 95% CI** |
| --- | --- | --- | --- |
| **Sample composition** |  |  |  |
| Total analytical sample | 422 | — | 92.3% retention |
| Male | 256 | 60.7% | — |
| Female | 166 | 39.3% | — |
| Saudi national | 142 | 33.6% | — |
| Non-Saudi international | 280 | 66.4% | — |
| Preclinical phase | 279 | 66.1% | — |
| Clinical phase | 143 | 33.9% | — |
| Fluent/conversational Arabic | 306 | 72.5% | — |
| Basic/no Arabic proficiency | 116 | 27.5% | — |
| Prior Saudi residence ≥3 years | 390 | 92.3% | — |
| **Primary outcome** |  |  |  |
| UWES-9S overall (0–6) | 4.15 | 0.84 | [4.07, 4.23]; 1.33–6.00; α = 0.82 |
| — Vigor | 4.36 | 0.95 | [4.27, 4.45]; α = 0.79 |
| — Dedication | 3.95 | 1.12 | [3.84, 4.06]; α = 0.81 |
| — Absorption | 4.14 | 1.18 | [4.03, 4.25]; α = 0.74 |
| **Main predictor** |  |  |  |
| BRS resilience (1–5) | 2.59 | 0.76 | [2.51, 2.65]; α = 0.79 |
| **Secondary predictor** |  |  |  |
| DSSI social support (10–30) | 23.96 | 5.69 | [23.44, 24.48]; α = 0.85 |

**SUPPLEMENTARY TABLE S2. Missing Data Analysis (N = 457 → 422)**

| **Variable** | **N Missing** | **% Missing** | **Mechanism** | **Action** |
| --- | --- | --- | --- | --- |
| Engagement (UWES-9S) | 2 | 0.4% | MCAR | Listwise deletion |
| Resilience (BRS) | 1 | 0.2% | MCAR | Listwise deletion |
| Social support (DSSI) | 3 | 0.7% | MCAR | Listwise deletion |
| Sex | 2 | 0.4% | MCAR | Listwise deletion |
| Prior residence | 5 | 1.1% | MCAR | Listwise deletion |
| **Total** | **35** | **7.6%** | **MCAR/MAR** | **Retained 92.3%** |

*Note: Little’s MCAR test χ² = 18.3, p = 0.14.*

**SUPPLEMENTARY TABLE S3. Sensitivity Analysis—Listwise vs. Multiple Imputation (N = 422)**

| **Predictor** | **Listwise β** | **MI β** | **Δβ** |
| --- | --- | --- | --- |
| Resilience | 0.418 | 0.421 | +0.003 |
| Language proficiency | 0.370 | 0.368 | −0.002 |
| Prior residence | 0.345 | 0.347 | +0.002 |
| Sex (female) | −0.410 | −0.408 | +0.002 |
| Social support | 0.104 | 0.106 | +0.002 |
| Clinical phase | −0.004 | −0.003 | +0.001 |
| **R²** | 0.528 | 0.530 | +0.002 |

**SUPPLEMENTARY TABLE S4. Regression Assumptions Testing (N = 422)**

| **Assumption** | **Test** | **Statistic** | **Status** | **Remedial Action** |
| --- | --- | --- | --- | --- |
| Multicollinearity | VIF | Range 1.26–1.84 | Passed | None |
| Heteroscedasticity | Breusch–Pagan | χ² = 12.4, p = 0.008 | Violated | HC3 robust SEs |
| Normality | Shapiro–Wilk | W = 0.992, p = 0.087 | Passed | None |
| Linearity | Partial regression plots | Linear | Passed | None |
| Independence | Durbin–Watson | 1.94 | Passed | None |
| Influential outliers | Cook’s distance | Max = 0.087 | Passed | None |

**SUPPLEMENTARY TABLE S5. Variance Inflation Factors by Model (N = 422)**

| **Predictor** | **Model 1** | **Model 4** | **Model 5** |
| --- | --- | --- | --- |
| Resilience | — | 1.38 | 1.42 |
| Clinical phase | — | 1.29 | 1.31 |
| Language proficiency | — | 1.35 | 1.39 |
| Prior residence | — | 1.51 | 1.54 |
| Social support | — | 1.22 | 1.24 |
| Sex (female) | — | 1.84 | 1.87 |
| **Mean VIF** | — | **1.43** | **1.46** |
| **Maximum VIF** | — | **1.84** | **1.87** |

**SUPPLEMENTARY TABLE S6. Ten-Fold Cross-Validation Results (Model 4)**

| **Metric** | **Mean (SD)** | **95% CI** |
| --- | --- | --- |
| Test R² | 0.596 (0.128) | [0.500, 0.691] |
| RMSE | 0.541 (0.095) | — |
| MAE | 0.414 (0.083) | — |

**SUPPLEMENTARY TABLE S7. Hierarchical Regression Models 1–3 (N = 422)**

| **Predictor** | **Model 1 β [95% CI]** | **Model 2 β [95% CI]** | **Model 3 β [95% CI]** |
| --- | --- | --- | --- |
| Resilience | 0.493*** [0.438, 0.549] | 0.454*** [0.397, 0.511] | 0.454*** [0.397, 0.511] |
| Clinical phase | — | −0.004 [−0.081, 0.073] | −0.004 [−0.081, 0.073] |
| Language proficiency | — | 0.355*** [0.269, 0.441] | 0.355*** [0.269, 0.441] |
| Prior residence | — | 0.360*** [0.258, 0.462] | 0.360*** [0.258, 0.462] |
| Social support | — | — | 0.004 [−0.065, 0.073] |
| **R²** | 0.348 | 0.483 | 0.483 |
| **ΔR²** | — | 0.135*** | 0.000 |
| **F (df)** | 225.3*** (1,420) | 97.7*** (4,417) | 77.4*** (5,416) |

**SUPPLEMENTARY TABLE S8. Primary Confirmatory Model 4 (N = 422)**

| **Predictor** | **β [95% CI]** | **f²** | **p** |
| --- | --- | --- | --- |
| Resilience | 0.418 [0.355, 0.481] | 0.370 | <0.001*** |
| Language proficiency | 0.370 [0.225, 0.515] | 0.267 | <0.001*** |
| Prior residence | 0.345 [0.231, 0.459] | 0.252 | <0.001*** |
| Sex (female) | −0.410 [−0.573, −0.247] | 0.356 | <0.001*** |
| Social support | 0.104 [0.025, 0.183] | 0.023 | 0.010** |
| Clinical phase | −0.004 [−0.083, 0.075] | 0.000 | 0.920 |
| **R²** | 0.528 |  |  |
| **Adjusted R²** | 0.520 |  |  |
| **F (df)** | 72.8*** (6,415) |  |  |

*Standardized coefficients; HC3 robust SEs applied.*

**SUPPLEMENTARY TABLE S9. Resilience × Clinical Phase Moderation Analysis (Model 5)**

| **Component** | **Value** | **95% CI** | **p** | **f²** |
| --- | --- | --- | --- | --- |
| **Model Fit** |  |  |  |  |
| R² | 0.565 | [0.535, 0.595] | <0.001 | --- |
| Adjusted R² | 0.558 | --- | --- | --- |
| F (df) | 73.1*** (7,414) | --- | <0.001 | --- |
| ΔR² (vs. Model 4) | 0.037 | --- | <0.001 | --- |
| **Interaction Term** |  |  |  |  |
| Resilience × Clinical Phase | 0.339 | [0.221, 0.457] | <0.001 | 0.129 |
| **Simple Slopes** |  |  |  |  |
| Preclinical students (Phase = 0) | 0.291 | [0.169, 0.413] | <0.001 | 0.061 |
| Clinical students (Phase = 1) | 0.630 | [0.460, 0.800] | <0.001 | 0.167 |
| **Amplification Factor** | 2.16× | --- | --- | --- |

*Note: Standardized coefficients reported. HC3 robust standard errors applied. Simple slopes represent resilience-engagement associations separately for preclinical and clinical training phases.*

**SUPPLEMENTARY TABLE S10. Gender-stratified regression models**

| **Predictor** | **Female β [95% CI]** | **Male β [95% CI]** |
| --- | --- | --- |
| Resilience | 0.41*** [0.315, 0.505] | 0.42*** [0.345, 0.495] |
| Clinical phase | 0.01 | −0.01 |
| Language proficiency | 0.35*** | 0.40*** |
| Prior residence | 0.30** | 0.39*** |
| Social support | 0.06 | 0.14* |
| **R²** | 0.52 | 0.53 |

**SUPPLEMENTARY TABLE S11. Bivariate correlation matrix with means and standard deviations (N = 422)**

| **Variable** | **1** | **2** | **3** | **4** | **5** | **6** | **7** | **M** | **SD** |
| --- | --- | --- | --- | --- | --- | --- | --- | --- | --- |
| 1. Engagement | — |  |  |  |  |  |  | 4.15 | 0.84 |
| 2. Resilience | 0.590*** | — |  |  |  |  |  | 2.59 | 0.76 |
| 3. Clinical phase | 0.050 | 0.127* | — |  |  |  |  | 0.34 | 0.47 |
| 4. Language proficiency | 0.376*** | 0.289*** | 0.042 | — |  |  |  | 0.73 | 0.45 |
| 5. Prior residence | 0.456*** | 0.186** | 0.089 | 0.314*** | — |  |  | 0.92 | 0.27 |
| 6. Social support | −0.127** | 0.156** | −0.031 | 0.087 | 0.045 | — |  | 23.96 | 5.69 |
| 7. Sex (0=M,1=F) | −0.086 | −0.091 | −0.043 | −0.063 | 0.027 | −0.178** | — | 0.39 | 0.49 |

***p < 0.001; **p < 0.01; *p < 0.05*

**SUPPLEMENTARY TABLE S12. Power analyses for primary, secondary, and exploratory models**

| **Analysis** | **Effect** | **Required N** | **Achieved power** |
| --- | --- | --- | --- |
| Main model | f² = 0.15 | ≥118 | >0.99 |
| Resilience × phase | f² = 0.129 | ≥230 | 0.85 |
| Exploratory interactions | — | — | 0.60–0.72† |

†Underpowered; exploratory only.

**SUPPLEMENTARY TABLE S13: Comprehensive Effect Size Summary Across All Analyses (n = 422)**

| **Effect Category** | **Effect/Predictor** | **Value** | **95% CI** | **p** | **Magnitude** |
| --- | --- | --- | --- | --- | --- |
| **BIVARIATE CORRELATIONS** |  |  |  |  |  |
|  | Resilience → Engagement | r = 0.590 | [0.525, 0.647] | <0.001 | Large (35% var) |
|  | Prior Residence → Engagement | r = 0.456 | [0.375, 0.529] | <0.001 | Large (21% var) |
|  | Language → Engagement | r = 0.376 | [0.291, 0.457] | <0.001 | Medium (14% var) |
| **PRIMARY MODEL EFFECTS (TABLE 3A)** |  |  |  |  |  |
|  | Resilience | f² = 0.370 | — | <0.001 | Large |
|  | Female Sex | f² = 0.356 | — | <0.001 | Large |
|  | Language Proficiency | f² = 0.267 | — | <0.001 | Medium |
|  | Prior Residence | f² = 0.252 | — | <0.001 | Medium |
|  | Social Support | f² = 0.023 | — | 0.010 | Small |
|  | Clinical Phase | f² = 0.000 | — | 0.920 | Negligible |
| **SECONDARY ANALYSIS (TABLE 3B)** |  |  |  |  |  |
|  | Resilience × Clinical Phase | f² = 0.129 | — | <0.001 | Medium-Large |
|  | Clinical slope amplification | 2.16× | — | — | Substantial |
| **SUBSCALE EFFECTS (TABLE 4)** |  |  |  |  |  |
|  | Resilience → Dedication | f² = 0.422 | — | <0.001 | Large |
|  | Resilience → Absorption | f² = 0.155 | — | <0.001 | Medium |
|  | Resilience → Vigor | f² = 0.108 | — | <0.001 | Medium |
|  | Clinical Phase → Dedication | f² = 0.136 | — | <0.001 | Medium |
|  | Clinical Phase → Absorption | f² = 0.123 | — | <0.001 | Medium |
|  | Social Support → Dedication | f² = 0.012 | — | <0.001 | Small |

*Note: This table presents selected effect sizes of primary theoretical importance. Complete subscale regression results with all predictors are available in Main Manuscript Table 4. Effect size interpretation follows Cohen (1988): small (f² ≥ 0.02), medium (f² ≥ 0.15), large (f² ≥ 0.35); correlations: small (r ≥ 0.10), medium (r ≥ 0.30), large (r ≥ 0.50).*

**Supplementary Table S14. Sensitivity analysis of the primary confirmatory model after excluding prior Saudi residence (≥3 years) as a predictor.**

| **Metric / Predictor** | **Main model (Table 3, Panel A) Std. β** | **Reduced model (without prior residence) Std. β** | **Δβ (Reduced − Main)** | **Significance pattern** |
| --- | --- | --- | --- | --- |
| **Model R²** | 0.528 | 0.525 | −0.003 | Unchanged overall fit pattern |
| **Adjusted R²** | 0.520 | 0.519 | −0.001 | Unchanged |
| Resilience (BRS) | 0.418 | 0.422 | +0.004 | Significant in both models |
| Language proficiency (fluent/conversational) | 0.370 | 0.370 | 0.000 | Significant in both models |
| Sex (female) | −0.410 | −0.411 | −0.001 | Significant in both models |
| Clinical training phase | −0.004 | −0.004 | 0.000 | Non-significant in both models |
| Social support (DSSI) | 0.104 | 0.099 | −0.005 | Significant in both models |

***Note:*** *Main model includes prior residence (≥3 years in Saudi Arabia). Reduced model excludes prior residence only. All other covariates, coding, and HC3 robust estimation are unchanged from the primary analysis.*
